# An injectable non-cross-linked hyaluronic-acid gel containing therapeutic spheroids of human adipose-derived stem cells

**DOI:** 10.1038/s41598-017-01528-3

**Published:** 2017-05-08

**Authors:** Jingwei Feng, Kazuhide Mineda, Szu-Hsien Wu, Takanobu Mashiko, Kentaro Doi, Shinichiro Kuno, Kahori Kinoshita, Koji Kanayama, Rintaro Asahi, Ataru Sunaga, Kotaro Yoshimura

**Affiliations:** 1 Department of Plastic Surgery, University of Tokyo, School of Medicine, 7-3-1, Hongo, Bunkyo-Ku, Tokyo, 113-8655 Japan; 2 Department of Plastic surgery, Southern Medical University Nanfang Hospital, 1838, Guangzhou South Ave., Guangzhou, 510515 China; 3 Department of Plastic Surgery, Tokushima University, School of Medicine, 3-18-15, Kuramoto-cho, Tokushima-shi, Tokushima, 770-8503 Japan; 4 Department of Plastic Surgery, Jichi Medical University, 3311-1, Yakushiji, Shimotsuke, 329-0498 Tochigi Japan

## Abstract

For chronic wounds, the delivery of stem cells in spheroidal structures can enhance graft survival and stem cell potency. We describe an easy method for the 3D culture of adipose-derived stem/stromal cells (ASCs) to prepare a ready-to-use injectable. We transferred suspensions of monolayer-cultured ASCs to a syringe containing hyaluronic acid (HA) gel, and then incubated the syringe as a 3D culture vessel. Spheroids of cells formed after 12 h. We found that 6 × 10^6^ ASCs/ml in 3% HA gel achieved the highest spheroid density with appropriate spheroid sizes (20–100 µm). Immunocytology revealed that the stem cell markers, NANOG, OCT3/4, SOX-2, and SSEA-3 were up-regulated in the ASC spheroids compared with those in nonadherent-dish spheroids or in monolayer cultured ASCs. In delayed wound healing mice models, diabetic ulcers treated with ASC spheroids demonstrated faster wound epithelialization with thicker dermis than those treated with vehicle alone or monolayer cultured ASCs. In irradiated skin ulcers in immunodeficient mice, ASC spheroids exhibited faster healing and outstanding angiogenic potential partly by direct differentiation into α-SMA+ pericytes. Our method of 3D in-syringe HA gel culture produced clinically relevant amounts of ready-to-inject human ASC microspheroids that exhibited superior stemness *in vitro* and therapeutic efficacy in pathological wound repair *in vivo*.

## Introduction

About 1% of the population suffers from some form of chronic wound at any given time^1^. Chronic wounds require repeated interventions and are often refractory to traditional therapies, causing tremendous suffering for patients and presenting a challenge for clinicians. Delayed wound healing essentially results from incompetent “seed” (stem/progenitor cells) and/or unhealthy “soil” (bioactive factors and matrix components)^2^. Adipose-derived stem/stromal cells (ASCs) are recognized for their capability to differentiate into fat, bone, and cartilage^3^. There has been an exponential increase in the number of clinical trials utilizing human ASCs (hASCs) because of the immunomodulatory function, trophic function, multipotency, and simple acquisition of ASCs^4, 5^. ASCs displayed reparative and regenerating effects in preclinical and clinical studies, strongly suggesting that they play a critical role in healing/remodeling after soft-tissue injury^4–6^.

In model systems and regenerative therapy, the ASC culture evolves from a monolayer culture to sheet, spheroid, organoid, and, ultimately, large multiplexed systems, mimicking the body’s organization from cells to layered tissues or simple organs^7^. Monolayer-cultured cells are poorly retained in local transplantations, nullifying the therapeutic intent or resulting in unexpected stem cell behaviors^8–10^. On the other hand, evolved three dimensional (3D) culture systems help to build sophisticated multicellular constructs with extracellular matrix (ECM) and demonstrate better therapeutic efficacy^9, 10^, leading to a new trend in stem cell therapy^11–13^. Among the 3D culture systems, the spheroid is comparatively simple to prepare and well-studied. Although there are many methods for 3D culture and spheroid formation, only the methods with simple equipment, high output, and low cost can be clinically relevant. Force floating on non-adherent dishes (NAD) is the most frequently adopted method, but the products often include many large aggregations with severe central necrosis^10, 14^. Matrices of biomaterial help to form spheroids of relatively uniform size and can efficiently produce therapeutic spheroids^15, 16^. Hyaluronic acid (HA) is a naturally derived ECM component that is widely used as an injectable material by ophthalmologists, arthrologists, and cosmetic surgeons. Previously, we found that HA served as a fine matrix for ASC spheroid formation. Spheroids prepared using HA gel have superior plasticity, trophic effects, and therapeutic potential for promoting tissue regeneration^15^.

Using a syringe as a 3D culture vessel, we developed a new method to make ASC spheroids in a syringe filled with HA gel (*HA ASC spheroids*). The method is very simple and productive and requires no special effort to extract the spheroids from the HA gel for therapies. We performed *in vitro* analyses to assess the viability, size, and stemness of the ASCs produced. In addition, we performed *in vivo* experiments using chronic-wound models to test the therapeutic efficacy and elucidate how the ASC spheroids facilitate wound healing.

## Results

### Optimization of *HA ASC spheroid* formation via 3D culture in syringe

#### Cell viability

**Figure 1:**
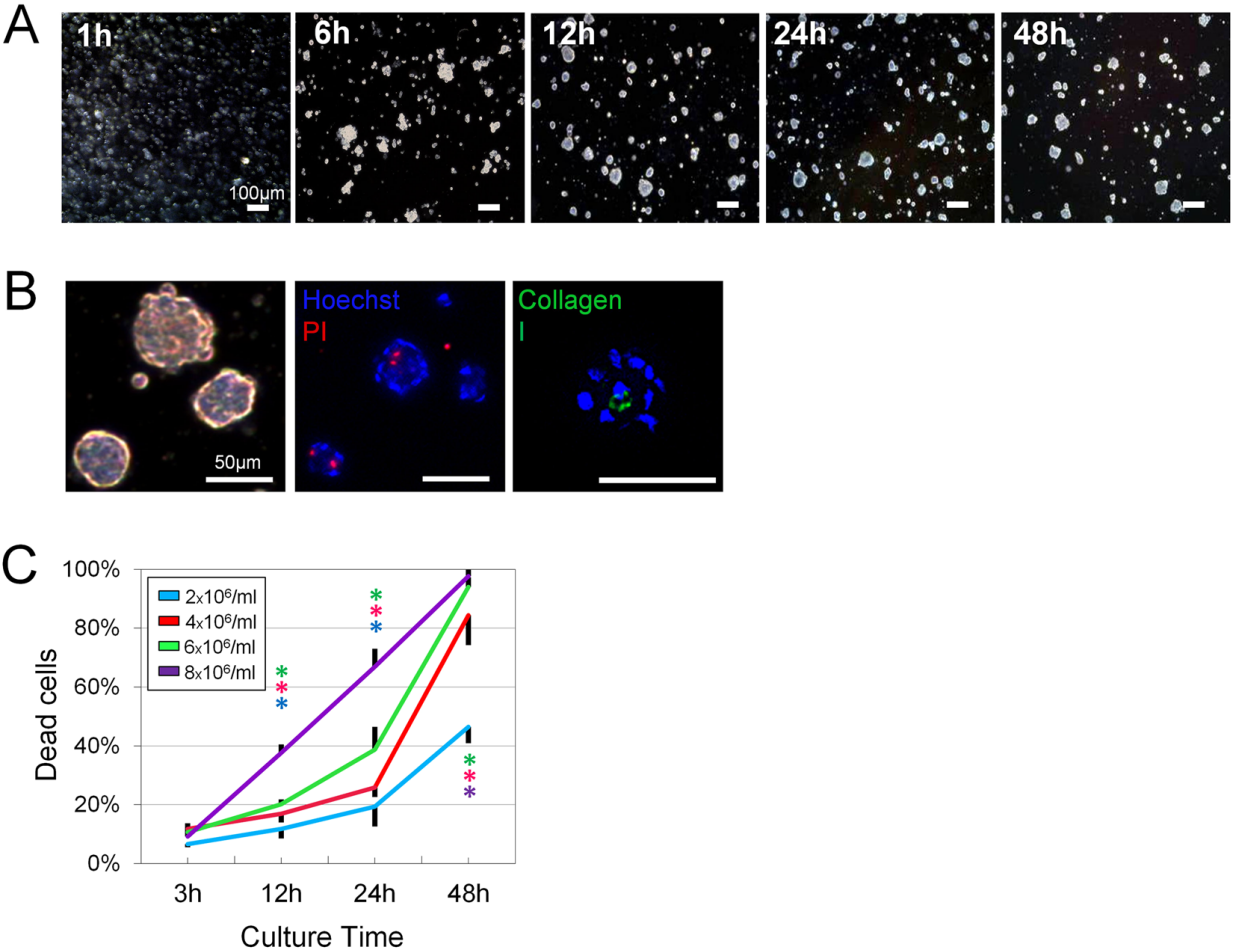
Optimization of spheroid formation and cell viability. (**A**) Temporal change of human ASC spheroid formation during in-syringe culture. Scale bars = 200 µm. (**B**) Light-microscopic images of spheroids after 12 h culture (left). All nucleate cells were labeled with Hoechst (blue), while dead cells were labeled with PI (red; middle). Spheroids expressed Collagen type 1 (green) in their center (right). Scale bars = 50 µm. (**C**) Quantification of dead cells produced by different seeding densities at different time points during in-syringe culture (n = 3 random fields of 500 × 700 µm; 500–1000 cells/field; *F = 49.48; *p* < 0.0001).

After seeding the ASCs in HA gel for 3D culture, we observed cellular aggregation as early as 6 h (Fig. 1A). The morphology of the spheroids (*HA ASC spheroids*) remained similar between 24 h and 48 h of culture, suggesting maturation of the spheroid formation (Fig. 1A). The cells were initially aggregated loosely, but spheroids with collagen type I expression became clearly margined at 12 h (Fig. 1A,B). We occasionally detected PI+ dead cells in the center of spheroids in the 12 h sample (Fig. 1B). Starting as early as about 24 h, the cell death rate increased logarithmically as the culture time progressed and the dead cells were seen not only in the center but also in the outboard of the spheroid (Fig. 1C). Severe cell death (>80%) occurred in the groups with higher ASC seeding densities (4 × 10^6^/ml, 6 × 10^6^/ml, and 8 × 10^6^/ml); however, in the 12 h sample, the death rate had not yet entered the logarithmic stage, and there was only 8.4% difference between the groups seeded with 2 × 10^6^/ml and 6 × 10^6^/ml ASCs (*p* < 0.01; M_2e6_ vs. M_6e6_, *p* < 0.05), respectively. The death rates in those groups ranged from 11.7% (±0.019) to 20.1% (±0.010). The group seeded with 8 × 10^6^/ml ASCs suffered from the most cell death (37.6% ± 0.029) at 12 h (M_2e6,4e6,6e6_ vs. M_8e6_, *p* < 0.01).

#### Spheroidal formative efficiency

**Figure 2:**
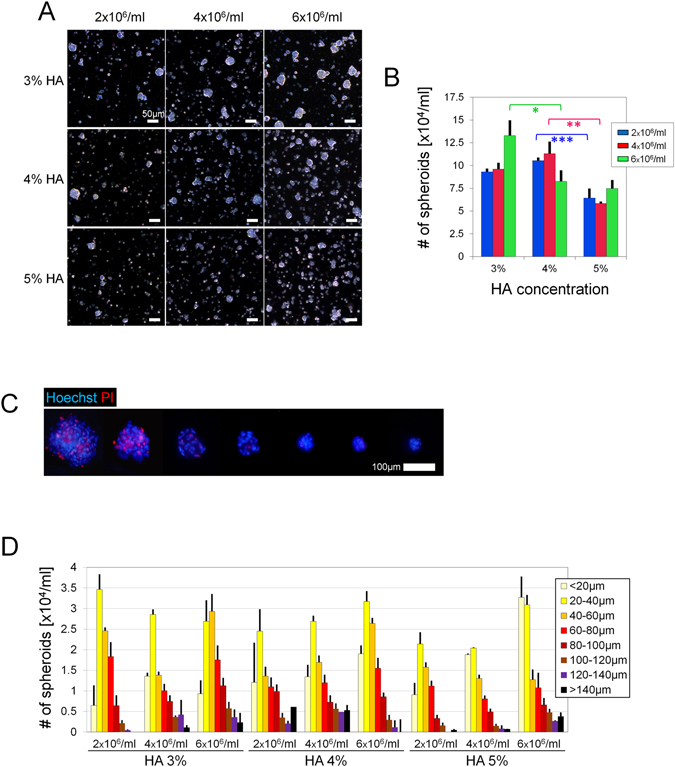
Optimization of spheroid preparation. (**A**) Representative microscopic images of spheroids after 12 h culture with different HA concentrations and seeding densities. Scale bars = 50 µm. (**B**) Total numbers of spheroids (>20 µm) yielded after 12 h culture. (**C**) Fluorescent microscopic images of spheroids of different sizes. Dead cells were labeled by PI (red) while nuclei were labeled with Hoechst (blue). Scale bars = 100 µm. (**D**) Size-distribution histogram of the products; n = 3 independent experiments; 50–200 particles of product analyzed in each sample; *p = 0.008, **p = 0.009, ***p = 0.034.

ASCs migrate less in thicker gels than in thinner gels. In our previous study^15^, HA gels thinner than 3% did not provide enough floatation, usually resulting in very large cellular aggregates at the gel bottom. At the other extreme, very thick HA gels prevented the gathering of cells. We found that 5% HA was the most concentrated gel that showed decent spheroid formation and was still able to be injected through a small (30 gauge) needle. Therefore, we chose 3%, 4%, and 5% HA as the concentrations for further testing. Based on the viability assays, we selected ASC concentrations of 2 × 10^6^/ml, 4 × 10^6^/ml, and 6 × 10^6^/ml. The higher seeding densities tended to produce higher numbers of spheroids, whereas the higher HA concentrations tended to produce smaller spheroids (Fig. 2A). Small particles (<20 µm) formed in every group, but the number of them should be minimized to improve the spheroidal formative efficiency. We found that the number of spheroids with size > 20 µm did not always increase as we put in more cells. In the group seeded with 6 × 10^6^/ml ASCs, the number of spheroids >20 µm decreased with the hardening of the HA gel with the presence of a large amount of undersized particles in the thick gel. In 3% HA, 6 × 10^6^/ml ASCs yielded 13.3 ± 1.21 × 10^4^ spheroids and seemed an optimal concentration for that density (*p* = 0.008) (Fig. 2B). In 4% HA, 4 × 10^6^/ml ASCs yielded the highest number of spheroids (11.3 ± 0.21 × 10^4^, *p* = 0.009). The total spheroid count did not, however, provide enough information to identify a single distinguished group.

#### Morphological analysis of HA ASC spheroids

Significant cell death appeared when the spheroid diameter reached approximately 100 µm (Fig. 2C). Assuming that the favorable size is 20–100 µm^17^, we calculated the numbers of spheroids that were undersized, oversized, and of favorable size to find the best combination of HA concentration and seeding density (Fig. 2D). There was a sophisticated relationship among gel concentration, cell number, and spheroid size. Although the higher seeding density might result in more spheroids, it could also promote the formation of larger spheroids and lead to a decreased number of spheroids. Likewise, the higher HA concentration could prevent the formation of large spheroids, thus increasing the number of microspheroids. The group with 3% HA and 6 × 10^6^/ml ASCs, produced the largest number of spheroids 20–100 µm (around 8.5 × 10^4^/ml). The peak of the size distribution lay in an even better range (40–60 µm). We therefore decided to prepare spheroids by seeding 6 × 10^6^/ml ASCs in 3% HA gel and to culture the cells for 12 h for further studies.

### Stem cell marker expression in HA ASC spheroids, NAD ASC spheroids and 2D cultured ASCs

**Figure 3:**
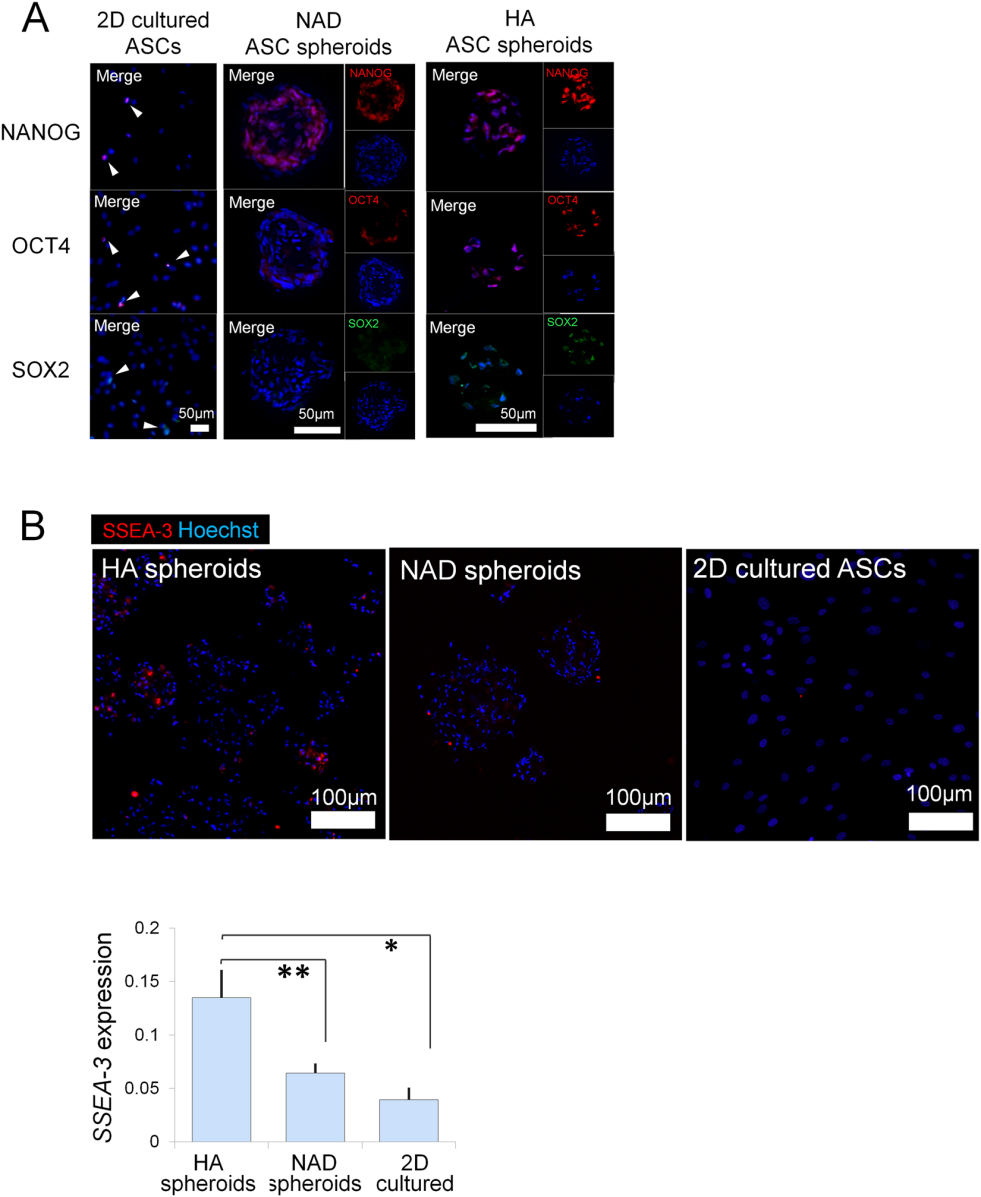
Stem cell marker expression of spheroids. (**A**) Immunohistology for nuclear stem cell markers, *NANOG*, *OCT4* and *SOX2*, in ASCs after 12 h culture on 2D petri dish, non-adhesive dish, and HA gel. Nuclei were labeled with Hoechst (blue). Scale bars = 50 µm. (**B**) SSEA-3 expression in ASCs 12 h after seeding on 2D petri dish (2D), non-adhesive dish (NAD), and in HA gel (HA). Bar graph showed the relative quantification of the SSEA-3 fluorescence. *p* = *0*.*019*, *^,^**p < 0.05; n = 3 independent experiments. Scale bars = 100 µm.

The immunohistocytology results indicated that spheroids prepared by 3D culture in HA gel (HA ASC spheroids) showed higher expression levels of NANOG, Oct-4, and SOX2 than 2D cultured ASCs or spheroids prepared with non-adherent dishes (NAD ASC spheroids) (Fig. 3A). NAD ASC spheroids expressed NANOG weakly only in the center part of the spheroid, leaving the outer layer of cells NANOG-negative. There was much stronger NANOG staining throughout the HA ASC spheroids. HA ASC spheroids showed significantly higher levels of SSEA-3, which is a marker for muse cells^18^, than NAD ASC spheroids (*p* = 0.043) and 2D cultured ASCs (*p* = 0.040) (Fig. 3B).

### Therapeutic effects of HA ASC spheroids in diabetic-mouse wound healing

**Figure 4:**
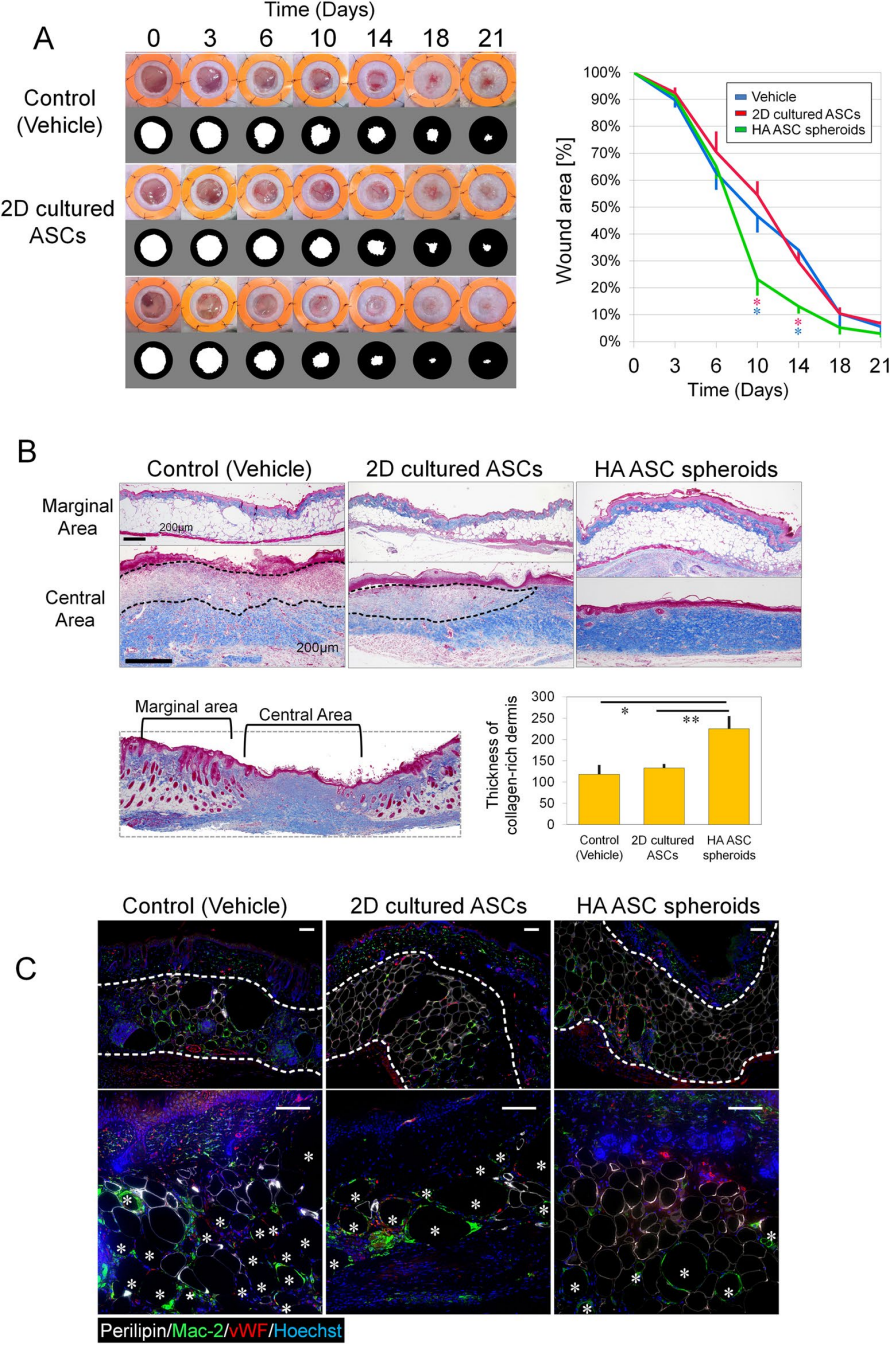
Wound healing of skin defects in diabetic mice. (**A**) Representative photos of wound healing in full-thickness cutaneous ulcers (left) and quantification of wound-opening sizes at different time points (right) in db/db mice. The inner diameter of the splints was 14 mm; n = 4 ulcers; *p_day10_ = 0.0245; *p_day14_ = 0.021. (**B**) Mallory-Azan trichrome stain of wound-tissue samples harvested 24 days post surgery (upper). Collagen was stained blue. Wound pathohistology was evaluated via quantification of dermal collagen deposition (lower). The dashed line indicates the collagen-poor area in the dermis; n = 4 ulcers; five points were photographically measured in each sample; *p = 0.040, **p = 0.043. Scale bars = 200 µm. (**C**) Immunofluorescence of the adipose layer of the wound: viable adipocytes (perilipin, white), macrophages (mac-2, green), vasculature (vWF, red), and nuclei (blue). *Represents crown-like structures which are perilipin-negative round area (oil drops) surrounded by Mac-2-positive infiltrated macrophages. Scale bars = 100 µm.

The mice injected with HA ASC spheroids healed more rapidly than those injected with 2D cultured ASCs or with vehicle (HA) alone (day 10: 31% smaller vs. 2D cultured ASC group; day 14: 17% smaller vs. 2D cultured ASC group; Fig. 4A). At week 3, wound closure was mostly completed in the HA ASC spheroid-treated mice, whereas the wound beds of mice treated with vehicle alone remained hyperemic.

We histologically evaluated the wound center and wound margin separately and found that scar tissue had formed in the central area (Fig. 4B **)**. Mallory-azan staining suggested that the subcutaneous adipose layer in the marginal area was thicker in the HA ASC spheroid-treated mice, while damaged adipocytes, forming large round oil droplets, were frequently seen in the adipose layer of vehicle-treated mice. In the HA ASC spheroid-treated mice, the normal dermis structure with abundant collagen fiber were seen. In the other mice (2D cultured ASC-treated or vehicle-treated), the scar tissue was seen as inflammatory collagen-deficient areas (dashed lines in Fig. 4B). The thickness of the collagen layer in the ulcer center indicated that the HA ASC spheroids promoted collagen deposition into the wound (*p* = 0.026).

Immunohistology indicated healthier subcutaneous tissue in the HA ASC spheroid-treated wounds (Fig. 4C). The HA ASC spheroid-treated wounds contained more viable adipocytes (perilipin+) than those treated with 2D cultured ASCs or with vehicle alone. The matured adipocytes were arranged in an orderly fashion in HA ASC spheroid-treated wounds, while there were also areas with some small (regenerating) adipocytes squashed together. In the dermis layer (marked by dashed lines in upper images of Fig. 4C), the HA ASC spheroid-treated wounds displayed well-developed microvasculature (vWF+) in the adipose inflammatory area, whereas the vehicle-treated wounds displayed less capillarization. The HA ASC spheroid-treated wounds contained fewer oil drops surrounded by Mac-2-positive macrophages forming “crown-like structures” (marked by*), indicating less inflammation and adipocyte necrosis (lower images of Fig. 4C).

### Therapeutic effects of HA ASC spheroids on wound healing in irradiated tissue

**Figure 5:**
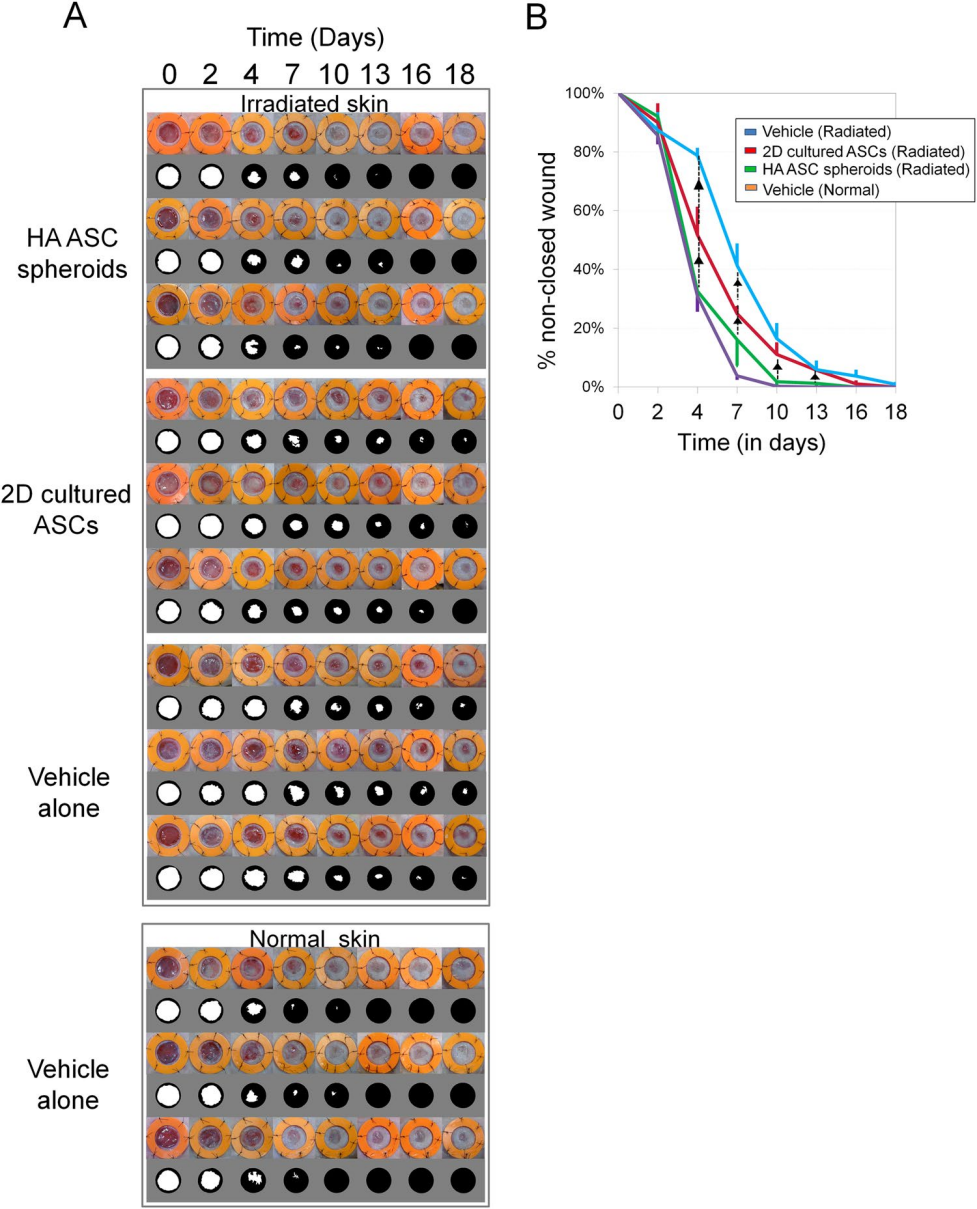
Macroscopic evaluation of wound healing in irradiated tissue of nude mice. (**A**) Sequential views of cutaneous ulcers (8 mm in diameter) treated with different cellular products or vehicle in irradiated or normal skin. The inner diameter of the splint was 14 mm. (**B**) Quantification of wound healing at different time points. n = 3 mice; *p*_day4_ = 0.0249, *p*_day7_ = 0.0249, *p*_day13_ = 0.0330, *p*_day16_ = 0.0364. ^▲^Represent *p* < 0.05.

The ulcers created in the irradiated tissue showed retarded healing compared with those in the normal tissue. By day 10, the vehicle-treated ulcers in normal tissue were essentially closed, while the vehicle-treated ulcers in irradiated tissue were left unhealed until day 18 (Fig. 5).

The HA ASC spheroid-treated irradiated ulcers, similarly to the vehicle-treated non-irradiated ulcers, showed better epithelialization and wound healing (wound area: 31.45%) than the 2D cultured ASC-treated irradiated ulcers (48.95%) by day 4, although the 2D cultured ASC-treated irradiated ulcers healed faster than the vehicle-treated irradiated ulcers (76.16%) (*H*_day4_ = *9*.*36*, *p*_day4_ = *0*.*0249*). The HA ASC spheroid-treated irradiated ulcers almost healed by day 10 (2.15%) or 13 (1.69%) (*p* < 0.05 for each pairwise comparison on days 10 and 13), whereas the other groups did not achieve full wound closure even on day 18.

**Figure 6:**
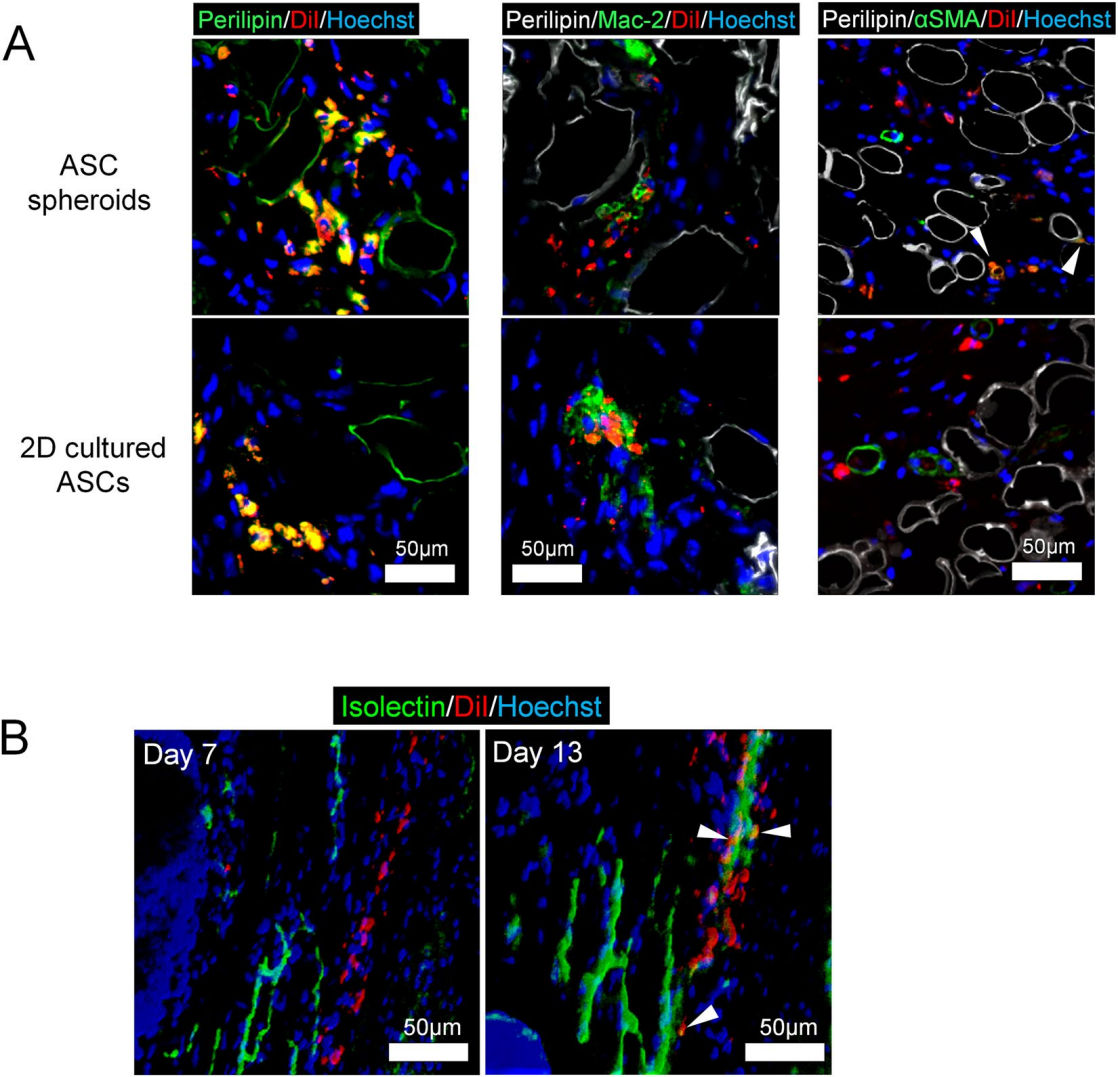
Immunohistological tracing of administered ASCs in irradiated skin ulcers of nude mice. (**A**) Immunohistological views of irradiated ulcer specimens 13 days after surgery and transplantation for tracing of administered ASCs (DiI-labeled cells). Cell fates were visualized by immunostaining for perilipin, Mac-2, and αSMA. Scale bars = 50 µm. (**B**) Whole mount staining of the irradiated wound tissue at days 7 and 13. Vascular endothelial cells were labeled by isolectin. Scale bars = 50 µm.

Immunohistology revealed the fate of administered 2D cultured ASCs or HA ASC spheroids which were labeled with DiI (Fig. 6A). On day 13, many perilipin(+)/DiI(+) cells (yellow cells) were detected in both HA ASC spheroid-treated and 2D cultured ASC-treated groups, which suggested that some administered ASCs started to differentiate into adipogenic lineages. Mac-2 immunostaining revealed that Mac-2(+) macrophages are located closely with DiI(+) ASCs, though the ASCs did not look expressing Mac-2. DiI(+) ASCs were frequently detected around vessels, though their exact localization and function remain unclear. DiI(+) ASCs, which differentiated into vascular mural cells and expressed αSMA, were occasionally seen in HA ASC spheroid-treated ulcers, but not in 2D cultured ASC-treated samples.

The whole-mount image of HA ASC spheroid-treated specimens revealed that DiI(+) ASCs arranged in a tubular structure along the isolectin(+) linear structure of endothelial cells in the dermis on day 7 (Fig. 6B). On day 13, DiI(+) tubular structures wrapping an isolectin(+) capillary were seen. Most of the DiI(+) cells laid around the capillaries, whereas DiI/isolectin double-positive cells were also detected occasionally.

## Discussion

Researchers have made many attempts to find ways to enhance mesenchymal stem cell (MSC) therapy for tissue repair. Delivery in spheroids was reported to enhance the cell stemness, differentiation/engraftment, and local retention^9, 10, 16, 17, 19^. Those beneficial effects were attributed to the cell-matrix microenvironment within the spheroids, which provides physiological/functional condition, mechanical support, and migration control^20^. Compared with other spheroid-preparation methods, our method does not involve any complicated tools or biomimetic materials and requires no spheroid-retrieving procedure for therapeutic use. Our method also increases the ASC loading number by more than one order of magnitude, from 3–7.5 × 10^4^/cm^2^ ^9, 10^ or 2.5 × 10^5^/ml^15^ to 10^6^/ml. That increase could allow physicians to process large quantities of cells using a less demanding procedure. Furthermore, the spheroids and matrix HA can be easily injected to a target organ/tissue through needles as fine as 27–30 gauge.

Because of a limit to the diffusion, cells in the center of large-sized spheroids suffer from hypoxia and malnutrition, leading to central necrosis^10, 21, 22^. Central necrosis was reported when spheroids of mesenchymal stem cells reached a diameter of 200 µm in a suspension rocking-culture system^23^. In this study, HA ASC spheroids produced central cell death at sizes exceeding 100 µm. Therefore, we set the size upper bound to 100 µm.

One of the advantages of spheroids is that they unlikely migrate too much because of their large size. Low retention of locally administrated cells has been reported as a crucial obstacle to accomplishing functional benefits^14, 16, 24^. Free cells injected into subcutaneous tissue could enter the lymph capillaries and travel through the lymph system. Clinical evidence of stem cell migration, such as ectopic lymphadenopathy, was found in patients that received local injections of cell suspensions^8^. The smallest lymphatic vessels (not capillaries) reside in the subcutaneous tissue, with typical diameters around 20 µm^25, 26^. We chose 20 µm as the lower bound that could theoretically prevent substantial migration.

It has been postulated that ASCs initiate or enhance skin wound repair by differentiating into skin cells; however, only a very small amount of ASCs can be integrated into the regenerated tissue^9, 18, 27^. In our experiments, the expression of pluripotency markers (NANOG, OCT-4, SOX2, and SSEA-3) was enhanced after 3D culture in HA gel. Previously, many stem cell markers were found to be elevated at the RNA level as well as the protein level^15^. The physiological microenvironment of stem cells is considered to regulate the stemness by enhancing NANOG and OCT-4^26^. The spheroidal structure mimics the physiological niche environment and may help to maintain the cells in an undifferentiated stage^9, 16, 28, 29^. The HA substrate was also reported to help embryonic stem cells (ESCs) remain undifferentiated *in vitro* and *in vivo* ^30, 31^. In some cancer cell lines, HA activates the major receptor CD44 and promote the expression of NANOG^32^, OCT-4, and SOX2^33^. Evidence has shown that by binding CD44, HA promotes Rho/ROCK signaling and enhances the stemness of MSCs^16^.

Another possible cause of the elevated ESC markers is hypoxia. It could be induced by spheroidal structure and high-density culture gel environment. We have learned that the spheroid center has a lower oxygen tension than the outer layers^10, 14^, and hypoxia can mediate or regulate the expression of NANOG and OCT-4^34–36^. In HA ASC spheroids, we found a NANOG-negative surface zone and a NANOG-positive core zone of spheroids (Fig. 3 A-middle), suggesting NANOG expression may correlate with oxygen level created by innate structure of spheroid. The high-density culture may further deteriorate the hypoxia, leading to ESC marker upregulation or cell death^37^. Further studies will be necessary, however, to explore the mechanisms by which the ASC-HA interaction and microenviromental cues enhance the ASC potency. Although HA ASC spheroids have elevated ESC markers, their mesenchymal markers such as CD90 persist indiscriminately^15^, which suggests that HA ASC spheroids do not raise ESC-like safety concern.

Multilinage differentiating stress enduring (MUSE) cells, marked by SSEA-3, show superior multi-/pluri-potency compared with regular MSCs^38^. MUSE cells made up only about 1% of 2D cultured ASCs and were expanded only in 3D culture^38, 39^, including spheroidal culture in HA gel^15^. Our HA ASC spheroids contained more SSEA-3+ cells than the NAD ASC spheroids and 2D cultured ASCs, which may help to show better therapeutic capacities.

We tested to explore the therapeutic potential in chronic wounds in ischemic conditions using two distinct animal models. In our diabetic-ulcer mice model, human HA ASC spheroids helped the wounds heal faster in the proliferative/regenerative stage of healing, although long-term retention of cells was not expected. In the wound periphery, the dermis showed signs of injury including severe inflammatory-cell infiltration and macrophage-surrounded dead adipocytes (crown-like structure). The HA ASC spheroids promoted successful repair of adipose tissue and abundant angiogenesis in the regenerating zone. In the wound center, which was already epithelialized, there was a neat and ordered collagen layer in the wounds treated with HA ASC spheroids. The other two treatments (2D cultured ASC-treated and vehicle-treated) resulted in delayed healing as well as inflamed and collagen-poor dermis. We reported previously that HA ASC spheroids secrete more growth factors in hypoxic conditions than monolayer-cultured cells^15^. Furthermore, SSEA-3(+) MUSE cells showed greater paracrine function and benefit to diabetic wound healing^40^. That might explain why the HA ASC spheroids accelerated and improved the healing of the diabetic ulcers in our db/db mouse model.

Another animal model is a nude mice with radiation-induced tissue damage. The cells, which are selectively killed by ionizing radiation, are dividing cells such as cancer cells and activated stem cells. The depletion of stem cells, stromal elements, and microvasculature thrombosis induced by hypoxia and ischemia caused late radiation damage and impaired healing competency^41^. The differentiation capacity and paracrine effects of ASCs can facilitate adipogenesis and angiogenesis, ameliorating the infertile condition of the irradiated tissue and improving wound healing. ASCs do not, however, usually incorporate into the vasculature if they are grafted as single cells^9, 10^. In contrast, in the HA ASC spheroid-treated radiated ulcers, we found that some of the transplanted ASCs became α-SMA+ in the dermis. Furthermore, those cells aligned in a tubular fashion before the end of the first week after transplantation and later appeared to become functional blood vessels. We believe that DiI-labeled ASCs from the HA ASC spheroids dissociated, migrated, and partly differentiated into pericytes and contributed to the restoration of vascular structures. The enriched SSEA-3+ MUSE cells in HA ASC spheroids might help for the superior differentiation and contribution to vascular formation.

## Conclusion

We investigated the therapeutic potential and limitations of ASCs delivered in suspension and developed a new, simple, 3D culture method using HA as a culture matrix. In only 12 h, in-syringe cultures of human ASCs can yield ready-to-inject cellular spheroids. The spheroids are relatively homogenous in size (20–120 μm) and express higher levels of stem cell markers than spheroids from non-adherent dish cultures. In-syringe culture allows clinicians to work with millions of cells using only a few simple procedures, which avoids loss of cellular viability and recovery. ASC spheroids prepared from culture in HA gels demonstrated superior efficacy in both refractory wounds created in diabetic mice and irradiated tissue in immunodeficient mice, which suggested both paracrine effects and functional differentiation.

## Materials and Methods

All experimental protocols involving human subjects in this study were performed in accordance with relevant government guidelines and approved by the institutional review board (IRB) of the University of Tokyo Hospital. were performed in accordance with the relevant guidelines and regulations. Prior to the procedure, each patient provided her informed consent using an IRB approved protocol. The protocols of animal experiments followed the relevant guidelines and were approved by the animal experimental committee of University of Tokyo School of Medicine.

### Cell isolation and culture

Liposuction aspirates were obtained from 6 healthy non-obese female donors aged 25–57 undergoing liposuction of the abdomen or thighs. Human ASCs were isolated from the aspirated fat as described previously^3^. Briefly, the aspirated fat was washed with phosphate-buffered saline (PBS) and digested on a shaker at 37 °C in PBS containing 0.075% collagenase for 30 min followed by centrifugation (700 g, 5 min). The pellets were rinsed, filtered and resuspended. The suspension was disseminated and cultured at 37 °C in a humid 5% CO_2_ atmosphere. The culture medium was Dulbecco’s Modified Eagle Medium (DMEM) supplemented with 10% fetal bovine serum (FBS). Primary cells were cultured for 7 days and were defined as passage 0.

### 3D culture of hASC spheroids with HA gel

**Figure 7:**
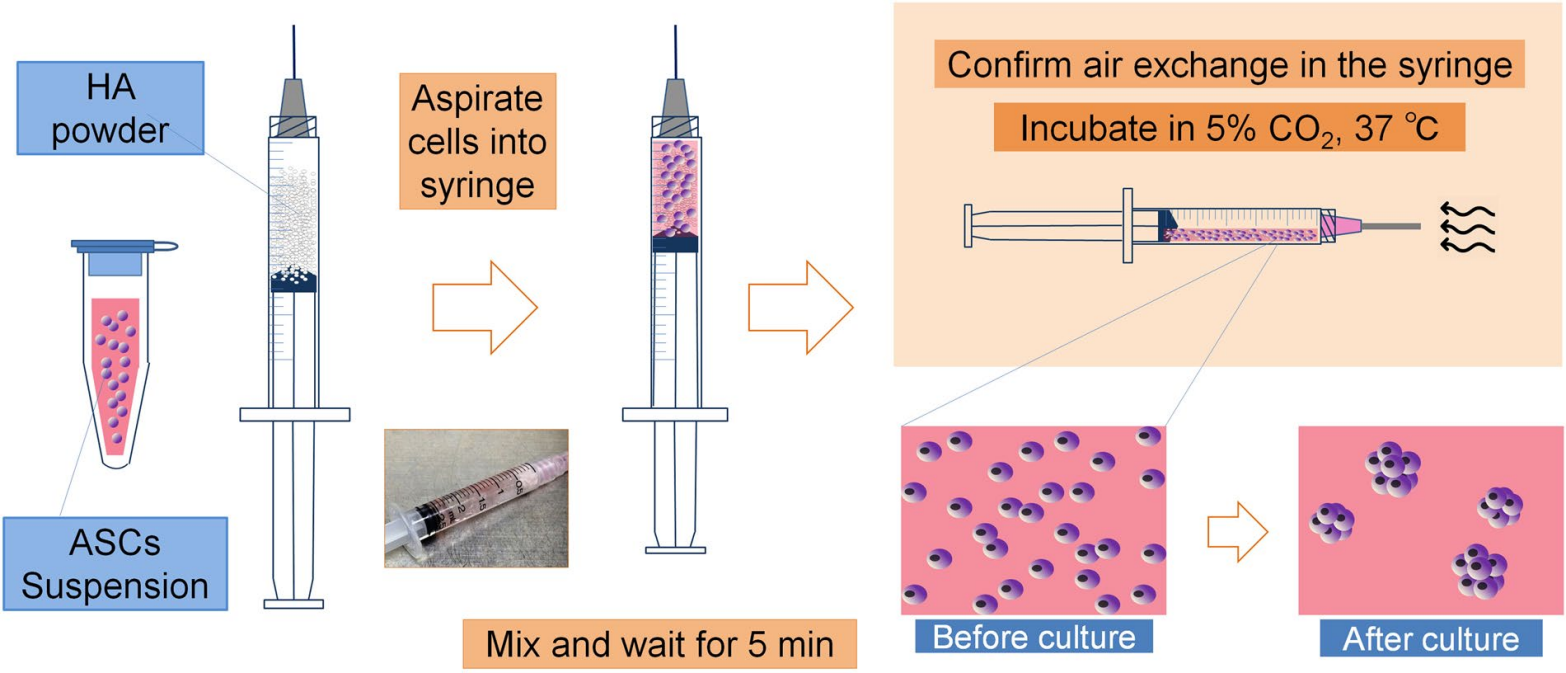
ASC spheroid formation in HA gel. Schematic diagram of the preparation of ready-to-inject HA spheroids. HA powder was weighed and transferred to a syringe. Monolayer-cultured ASCs were trypsinized, counted, suspended in growth medium, aspirated into the syringe, and mixed with the powder, which was then allowed to dissolve for 10 min. The plunger was drawn back to assure air exchange through the needle. The needle cap then closed, and the syringe was incubated at 5% CO_2_ and 37 °C. The gel slowly sank to the lower half of the syringe.

We weighed non-cross-linked sodium hyaluronate powder (average molecular weight of 1,000 kDa, Kikkoman Biochemifa Company, Tokyo, Japan, http://biochemifa.kikkoman.co.jp) inside a tissue-culture hood using a 2.5 ml luer-lock syringe as a weighing tube. As the HA powder was a sterilized (pharmaceutical grade) product, no special sterilization was needed for preparation of the HA gel. After transferring the desired amount of HA powder to the syringe [e.g., 30 mg HA for preparing 3% (w/v) HA gel of 1 ml in volume], we trypsinized and counted monolayer-cultured, first-passage or second-passage hASCs, resuspended the desired amount of cells in 1 ml DMEM supplemented with 10% FBS, aspirated the cell suspension into the syringe containing the HA powder, and inverted the syringe several times to mix the contents. The mixture became sticky gel over time depending on the concentration of HA powder. The cell-containing HA gel became homogenous without any additional culture medium. Later, we created some air space within the syringe by drawing the plunger back and replaced the original needle with an 18-gauge needle. Partial tension of oxygen (*p*O_2_) tension of the fluid in the syringe was measured with an oxygen monitor using a wire electrode (Eiko Kagaku, Tokyo, Japan). After confirming normoxia in the syringe, we laid the syringe flat in a 37 °C and 5% CO_2_ incubator (Fig. 7). We harvested the culture products at several different time points for further analysis.

### Dead cell quantification in hASC spheroids

We conducted a viability assay in order to optimize the in-syringe culture time and seeding density. We used DMEM with 1% Hoechst (v/v) and 0.5% propidium iodide (v/v) (PI) to label the cell nuclei and dead cells, respectively. We sampled and stained the cellular products at 37 °C for 10 min and took photographs with a fluorescence microscope (Keyence, Osaka, Japan). We performed “Z-stack” scans of 200 µm thickness and quantified using the BZ-II analyzer software (Keyence). n = 3 independent experiments, each includes random fields of 500 × 700 µm; 500–1000 cells/field.

### Spheroid count and morphological analysis of hASC spheroids

After a series of preliminary tests, we chose 3%, 4%, and 5% HA gel to prepare hASC spheroids. For each concentration, we seeded 2 × 10^6^, 4 × 10^6^, and 6 × 10^6^ cells and cultured the cells for 12 h. For spheroid number count, we extracted spheroids from HA by saline washing to include more particles and took phase-contrast photographs. Each sample of the same volume included 50–200 particles over 20 μm. For spheroid size measurement, we took phase-contrast photos of the original products to achieve best measurement accuracy by a software. Each sample of the same volume included 50–200 particles. We used the two different BZ-II analyzer softwares to measure the particle number and size, respectively. We performed PI staining to visualize the dead cells in spheroids of different sizes. Size distribution analysis included 3 independent experiments; 50–200 particles of product analyzed in each sample.

### Immunocytology of hASC spheroids

We harvested the hASC spheroids prepared in HA gels (HA ASC spheroids) and compared them with spheroids made using a non-adherent dish (NAD ASC spheroids). We used 3D microtissue petri dishes (non-adherent) to make comparable spheroids of similar size to the spheroids in HA gel. We added 20,000 hASCs in 190 µl DMEM (10% FBS) to a single agarose 256-well microdish and harvested the cells after 12 h of floating culture. In addition, we prepared another group of monolayer-cultured ASCs (2D cultured ASCs) on culture slides. We used the harvested spheroid pellets to make frozen blocks. We cut and fixed sections 7 µm thick and then blocked the sections. We used the following primary antibodies: NANOG antibody [N3C3] (GeneTex, Irvine, CA), OCT4 antibody (GeneTex), SOX2 antibody (GeneTex), anti-SSEA-3 antibody (EMD Millipore, Darmstadt, Germany), and anti-collagen I antibody (Abcam, Cambridge, U.K.). We diluted the antibodies 1:200 (collagen type I antibody 1:500) and incubated them for 16 h at 4 °C. We then incubated the samples with secondary antibody of goat anti-rabbit IgG Alexa Fluor 594, goat anti-rabbit IgG Alexa Fluor 488, or goat anti-rat Alexa Fluor 594 with 1:200 dilution for 1 h and took photographs using a fluorescence microscope.

### Mallory-Azan trichrome stain and evaluation of collagen deposition

We fixed the wound samples, embedded them in paraffin, and cut 5 µm sections. We then stained the specimen with Mallory-Azan solution following manufacturer’s instruction. We then inspected the slides by microscopy (Keyence) and took images of the healed dermis. We identified the ulcer center as an adipose-free, muscle-free, and collagen-rich area. We measured the dermal thickness by dividing the blue (collagen) dermal area by the width of wound center zone using a BZ-II analyzer. We compared the dermal thickness between treatment groups.

### Four-color immunofluorescence

We cut the zinc-fixed paraffin blocks into 5 µm sections and deparaffinized, rehydrated, and incubated them in a container with target-retrieval solution (pH = 9) by boiling the container in water for 20 min. We then permeabilized the sections in 0.1% triton-X TBS and blocked them with 1% BSA in buffer for 1 h. We prepared primary and secondary antibody solutions in TBST (0.1% Tween 20, 1% BSA) with the antibodies listed in Table 1. We incubated the samples in the primary antibody solution for 16 h at 4 °C and then washed them with three changes of TBS. We diluted the secondary antibodies and Hoechst 200× in TBST and incubated them with the sections for 1 h, followed by another three changes of TBS, and mounting. We used a fluorescent microscope with the listed filters (Table 1 **)** to view the slides and take photographs.

**Table 1:** Materials used for immunofluorescence.

Primary antibody	Dilution	Secondary antibody/dye	Fluorescent filter
Guinea pig anti-Perilipin/PLIN1 (Progen, Heidelberg, Germany)	1:1000	Goat anti-guinea pig IgG, Alexa Fluor 647	CY5 EX620/60 DM660 BA700/75
Rabbit anti-von Willebrand factor (DAKO)	1:200	Goat anti-rabbit IgG, Alexa Fluor 594	TRITC EX545/25 DM565 BA605/70
Rat anti-MAC-2 (Cedarlane, Ontario, Canada)	1:1000	Goat anti-rat IgG Alexa Fluor 488	GFP EX470/40 DM495 BA525/30
N/A	N/A	Hoechst	DAPI EX360/40 M400 BA460/50

### DiI labeling of the hASC spheroids

We cultured first-passage hASCs on a petri dish with DMEM supplemented with 10% FBS, stained the cell suspension with 2 μM CM-DiI (Thermo Fisher Scientific) in Hank’s balanced salt solution (HBSS) at 37 °C for 5 min and then at 4 °C for 15 min, and plated them back onto the dishes. For the next 2 days, we repeated the staining procedure daily to obtain the optimal cellular fluorescence.

### Whole-mount staining and imaging

We euthanized the mice and harvested and embedded fresh wound-skin samples to make frozen blocks. We stained sections 20 µm thick with the following dye buffer: 200× Hoechst, isolectin GS-IB4, and Alexa Fluor 488 conjugate in HBSS buffer. We stained the sections for 30 min at 37 °C in the dark, washed them, and sent them directly for confocal microscopy (Leica, Wetzlar, Germany). We examined the subcutaneous layer, scanning a total of 15 µm tissue depth.

### Mouse diabetic-ulcer model

The care of B6-db/db mice (BKS.Cg/Leprdb/m/JCL, 8-week-old male) was conducted in accordance with institutional guidelines using a protocol approved by the Animal Experimental Committee of the University of Tokyo. We anesthetized the mice by isoflurane inhalation, depilated them, and created two full-thickness cutaneous wounds (8 mm diameteron both sides dorsally using a sterile circular biopsy punch. We then placed a donut-shaped silicone splint to prevent wound contraction. We directly injected 100 μl human ASC spheroids (prepared from 0.6 × 10^6^ ASCs, cultured in 3% HA gel for 12 hours; HA ASC spheroids) by 27-gauge needle into four different points of subcutis at each wound periphery (n = 4 ulcers). We injected the same number of monolayer cultured human ASCs grown on a normal petri dish (2D cultured ASCs) and mixed with 3% w/v HA gel in DMEM supplemented with 10% FBS into another group of mice. We used injections of 3% w/v HA gel in DMEM supplemented with 10% FBS as a vehicle (control). We covered the treated wounds with sterile silicone gauze and transparent sterile dressings. We photographed the wounds on days 0, 3, 6, 10, 14, 18, and 21. We identified the edges of the wound openings as the border of the whitish, dry, membrane-like structure and determined the areas using Photoshop CS5. After each photograph, we cleaned the wounds and changed the dressings.

### Mouse radiation-ulcer model

We purchased 12-week-old male nude mice (BALB/cAJcl-FOXN1^*nu*/*nu*^) from CLEA (Tokyo, Japan) and applied irradiation to the skin using an MX-160Labo X-ray radiation machine (Medixtec, Chiba, Japan). We anesthetized the mice by isoflurane inhalation on the radiation table, placed them in a lateral position, pulled the dorsal skin up to create a skin fold, and taped the skin to the radiation surface. We covered the body and tail with a lead shield, leaving the dorsal skin fold unprotected, and applied 10 Gy ionizing radiation. We then housed the mice separately for 4 weeks. We created an 8 mm full-thickness cutaneous ulcer on the back of each mouse as described above. There were four groups: irradiated mice with cutaneous ulcer treated by human ASC spheroids (product from 0.6 × 10^6^ hASCs, cultured in 3% HA gel for 12 hours; HA ASC spheroids), by the same number of monolayer cultured human ASCs (suspended in 3% HA gel in DMEM with 10% FBS; 2D cultured ASCs), or by vehicle (3% HA gel in DMEM with 10% FBS); and normal mice with cutaneous ulcer treated by vehicle (n = 3 mice/group); and normal mice with cutaneous ulcer treated by vehicle (n = 3 mice/group). We photographed the wounds on days 0, 2, 4, 7, 10, 13, 16, and 18.

### Statistical analysis

We expressed the results as the mean ± standard error. We used Kruskal-Wallis test to compare the wound sizes in both animal models. We applied one-way ANOVAs to the viability assay, the SSEA-3 quantification, and the comparison of scar-collagen thickness. Tukey’s HSD served as a post-hoc test. We considered *p*-values < 0.05 statistically significant.
